# Amelioration of Autoimmune Diabetes of NOD Mice by Immunomodulating Probiotics

**DOI:** 10.3389/fimmu.2020.01832

**Published:** 2020-09-03

**Authors:** Tae Kang Kim, June-Chul Lee, Sin-Hyeog Im, Myung-Shik Lee

**Affiliations:** ^1^Department of Internal Medicine, Severance Biomedical Science Institute, Yonsei University College of Medicine, Seoul, South Korea; ^2^Department of Health Sciences and Technology, SAIHST, Sungkyunkwan University School of Medicine, Seoul, South Korea; ^3^ImmunoBiome. Inc., Pohang, South Korea; ^4^Division of Integrative Biosciences and Biotechnology, Pohang University of Science and Technology, Pohang, South Korea

**Keywords:** probiotics, autoimmune diabetes, regulatory T cells, gut homing receptor, gut permeability

## Abstract

Type 1 autoimmune diabetes is an autoimmune disease characterized by specific destruction of pancreatic β-cells producing insulin. Recent studies have shown that gut microbiota and immunity are closely linked to systemic immunity, affecting the balance between pro-inflammatory and regulatory immune responses. Altered gut microbiota may be causally related to the development of immune-mediated diseases, and probiotics have been suggested to have modulatory effects on inflammatory diseases and immune disorders. We studied whether a probiotic combination that has immunomodulatory effects on several inflammatory diseases can reduce the incidence of diabetes in non-obese diabetic (NOD) mice, a classical animal model of human T1D. When Immune Regulation and Tolerance 5 (IRT5), a probiotic combination comprising *Lactobacillus acidophilus, Lactobacillus casei, Lactobacillus reuteri, Bifidobacterium bifidium*, and *Streptococcus thermophiles*, was administered 6 times a week for 36 weeks to NOD mice, beginning at 4 weeks of age, the incidence of diabetes was significantly reduced. Insulitis score was also significantly reduced, and β-cell mass was conversely increased by IRT5 administration. IRT5 administration significantly reduced gut permeability in NOD mice. The proportion of total regulatory T cells was not changed by IRT5 administration; however, the proportion of CCR9^+^ regulatory T (Treg) cells expressing gut-homing receptor was significantly increased in pancreatic lymph nodes (PLNs) and lamina propria of the small intestine (SI-LP). Type 1 T helper (Th1) skewing was reduced in PLNs by IRT5 administration. IRT5 could be a candidate for an effective probiotic combination, which can be safely administered to inhibit or prevent type 1 diabetes (T1D).

## Introduction

Type 1 autoimmune diabetes is a classical organ-specific autoimmune disease resulting from immune-mediated destruction of pancreatic β-cells producing insulin. While genetic predisposition plays a critical pathogenic role both in patients with T1D and in animal models of autoimmune diabetes, environmental factors are also important for the development of clinical disease.Among environmental factors, the microbiota is emerging as a crucial element that can influence T1D by modulating local and systemic host immunity. Particularly, gut microbiota represents the largest microbial population in humans or animals which affects not only immune responses but also other vital processes of life, such as nutrient uptake.

Currently, microbiota studies are gaining strong popularity since new methods of microbiota identification, such as 16s rRNA gene sequencing revolutionized the field. However, the role of microbiota in T1D has been long recognized as exemplified by the decade-old “hygiene hypothesis” (1). According to the hypothesis, recent increases in the incidence of autoimmune or allergic diseases including T1D could be due to improved sanitation and relative paucity of exposure to microorganisms that are necessary for the proper maturation or functional adaptation of the immune system (2). Microbial exposure can influence host immunity by multiple mechanisms including Th1/type 2 T helper (Th2) deviation or regulatory cell modulation (3). Consistently, when NOD mice, a classical model of autoimmune diabetes, were rendered germ-free, insulitis was accelerated which was accompanied by increased Th1 and Th17 cells in the mesenteric lymph nodes (MLNs) and PLNs (4); however, the incidence of diabetes was not significantly changed in germ-free NOD mice (4, 5), showing complex nature of the development of the disease phenotype *in vivo*. Regarding specific microorganisms that can influence autoimmune diabetes, *Lactobacillus johnsonii, Lactobacillus casei, Bacillus cereus, Akkermansia muciniphila, Segmented Filamentous Bacteria* (SFB), a specific strain of *Clostridium butyricum* or probiotic comprising such bacteria have been reported to reduce the incidence of diabetes in NOD mice or BB rat, a rat model of autoimmune diabetes by modulating cytokine profile, Treg cells, Th cell polarization, or barrier function (5–10). Viruses, such as norovirus or lymphocytic choriomeningitis virus (LCMV) have also been shown to inhibit the development of autoimmune diabetes in NOD mice (11, 12).

Because of profound effects of microbiota on host immunity and therapeutic potential, a large array of microbes or their products has been employed to treat or manage diverse inflammatory diseases, metabolic diseases and cancers (13–16). Particularly, probiotics, live microorganisms conferring a beneficial physiological effect on the host, such as maintenance of host immune homeostasis, nutrient metabolism or protection of neonates from infection (17–19), are attractive candidates as potential therapeutic agents against those diseases. We have recently reported a probiotic combination comprising 5 bacteria, IRT5, that has therapeutic effects on several autoimmune or inflammatory disease models, such as inflammatory bowel disease, atopic dermatitis, rheumatoid arthritis, and experimental autoimmune encephalomyelitis (20, 21). Here, we studied whether the microbial combination could be effective in autoimmune diabetes of NOD mice.

## Materials and Methods

### Animals

NOD mice and BDC2.5/NOD T cell receptor (TCR)-transgenic mice purchased from Jackson Laboratory (Bar Harbor, ME, USA) were maintained in a specific-pathogen-free environment in the vivarium of Yonsei University College of Medicine. Mice were considered diabetic if blood glucose levels were >16.7 mmol/l on a single measurement or > 13.9 mmol/l on consecutive measurements. The incidence of diabetes in female and male NOD mice was about 70 and 30%, respectively, at 24 weeks of age. All animals had free access to water and laboratory chow and were kept on a 12-h light/dark cycle. All mouse experiments were conducted in accordance with the Public Health Service Policy in Humane Care and Use of Laboratory Animals. Mouse experiments were approved by the IACUC of the Department of Laboratory Animal Resources of Yonsei University College of Medicine, an AAALAC-accredited unit.

### Probiotic Administration

IRT5 (kindly provided by Korea Yakult Co, Giheung, Korea) was mixtures of live bacteria consisting of 1 × 10^9^ colony-forming unit (cfu) of each of the following strains in 100 μl: *Lactobacillus acidophilus, Lactobacillus casei, Lactobacillus reuteri, Bifidobacterium bifidium*, and *Streptococcus thermophiles*. Four-weeks-old female NOD mice were randomly assigned to experimental group treated with IRT5 (NOD-IRT5) or control group treated with PBS (NOD-PBS). Mice were healthy and non-diabetic before starting IRT5 administration, and no adverse effect of IRT5 administration was observed. Study design and general scheme of probiotic administration are shown in Figure S1.

### Insulitis Scoring and β-Cell Mass

Insulitis score was determined using paraffin-embedded H&E stained sections as previously described (22). In brief, the severity of insulitis was evaluated from more than 30 pancreatic islets from 3 or more parallel sections of different cut levels per mouse. The degree of insulitis was categorized into 4 groups: 0, no insulitis; 1, periinsulitis with or without minimal lymphocytic infiltration into islets; 2, invasive insulitis with islet destruction of ≤ 50%; 3, islet destruction of > 50%. Relative β-cell mass was measured by point counting after insulin immunohistochemistry of pancreatic sections, as previously described (23). Insulitis scoring and β-cell mass determination were conducted after 12 weeks of IRT5 administration.

### Insulin Autoantibody (IAA) Measurement

Serum mouse IAA levels were determined using an ELISA kit (ABclonal, Woburn, MA, USA), according to the manufacturer's instructions. Briefly, 50 μl of enzyme solution was added to samples and standards in 96-well plates. After incubation for 1 h at 37°C in a humid chamber and washing, substrate was added for incubation for 15 min at room temperature without light exposure. After adding 50 μl of stop solution to each well, optical density (O.D.) was determined at 450 nm.

### Gut Permeability

Permeability of gut epithelium was determined as described (24). In brief, mice fasted for 4 h were orally administrated with 60 mg/100 g body weight permeability tracer of fluorescein isothiocyanate (FITC)-labeled dextran (molecular weight, 4,000) (Sigma, St. Louis, MO, USA) and peripheral blood was collected 4 h later. Serum samples were diluted in equal volumes of PBS, and the serum FITC-dextran concentrations were determined using a fluorescence spectrophotometer (GloMax-Multi Detection System) (Promega, Madison, WI, USA) at an excitation wavelength of 490 nm and an emission wavelength of 540 nm.

### Immunohistochemistry

For immunohistochemical staining of tight junction proteins, 2–4 μm thin sections of the small intestine (SI) were deparaffinized and rehydrated. After antigen retrieval by microwaving in Tris-EDTA buffer (10 mmol/l, pH 9.0) for 10 min and blocking in 10% goat serum for 30 min, sections were incubated with ZO-1 antibody (Ab) (Invitrogen, Carlsbad, CA, USA) for 30 min at room temperature. Sections were then incubated with biotinylated secondary antibody for 30 min, followed by incubation with Vectastain ABC (Vector Lab, CA, USA) and DAB (Invitrogen) staining. Between each step, sections were rinsed three times in TBST buffer (137 mM NaCl-20 mM Tris, pH 7.4–0.1% Tween 20). After DAB staining, sections were counterstained with Mayer's hematoxylin.

### T Cell Priming

T cell priming was evaluated by an adoptive transfer system using carboxyfluorescein succinimidyl ester (CFSE) (Molecular Probes, Eugene, OR, USA)-labeled lymphocytes as described (25). In brief, naïve CD4^+^ T cells were prepared from the pooled spleens of young BDC2.5/NOD mice by the negative-selection method using a MACS CD4^+^ T cell isolation kit (Miltenyl Biotech, Alburn, CA, USA). The purity of CD4^+^ T cells was > 95%. CFSE-labeled CD4^+^ T cells (2 × 10^6^ cells) were transferred into recipient mice by tail vein injection. Lymphoid cells in PLNs, MLNs and SI-LP were harvested 66 h after transfer, and single cell suspension was analyzed for CFSE dilution by flow cytometry gated on CD4^+^ and Vβ4^+^ cells.

### Flow Cytometry

Regulatory T (Treg) cell proportion was determined as published (16). In brief, single-cell suspensions of lymphocytes were manually prepared from PLNs, MLNs or SI-LP of mice. To harvest SI-LP cells, SI was opened and washed in ice-cold PBS. After washing with PBS, tissues were transferred to a flask containing 10% FBS-10 mmol/l EDTA in PBS. After vigorous shaking, tissues were passed through a strainer. Tissues were then minced in 10 ml RPMI 1640 containing 0.5 mg collagenase D (Roche, Indianapolis, IN, USA) and DNase I (Roche). After stirring for 20 min at 37°C, the supernatant was collected for a total of 3 times. Cell pellet obtained after centrifugation at 520 *g* for 5 min was washed and resuspended in RPMI-2% FBS. SI-LP mononuclear cells were purified on a 40–75% Percoll (GE Healthcare Life Sciences, Uppsala, Sweden) gradient by centrifugation at 930 *g*, 25°C for 20 min. Cells were resuspended in PBS-2% FBS-2 mM EDTA. After Fc blocking, PLNs, MLNs or SI-LP cells were incubated with a mixture of anti-CD3ε and -CD4 Abs (eBioscience, San Diego, CA, USA) in PBS-2% FBS-2 mM EDTA at 4°C for 30 min. After permeabilization, cells were incubated with anti-mouse/rat FOXP3 (FJK-16s) Ab (eBioscience) at 4°C for 30 min. The FoxP3 Staining Buffer Set (eBioscience) was used for intracellular staining according to the manufacturer's instructions.

To evaluate Th or cytotoxic T (Tc) cell skewing, cells in PLNs, MLNs, and SI-LP were stimulated immediately after isolation with phorbol 12-myristate 13-acetate (Sigma), ionomycin (Sigma) in the presence of BD GolgiStop (BD Biosciences) at 37°C for 4 h. After Fc blocking, cells were stained with fluorophore-labeled Abs specific for CD3ε, CD4, or CD8 (eBioscience) in PBS-2% FBS-2 mM EDTA at 4°C for 30 min. After surface staining, cells were stained with anti-interleukin 17 A (IL-17A) (eBioscience) and -IFN-γ Ab (eBioscience) at 4°C for 30 min using the Cytofix/Cytoperm kit (BD Pharmingen, San Diego, CA, USA), according to the manufacturer's instructions. Cells were fixed in 0.5% paraformaldehyde, and then multi-color flow cytometry was performed using a FACSCalibur flow cytometer (BD Biosciences, San Jose, CA, USA). Data were analyzed with FlowJo software (Tree Star, Inc., Ashland, OR, USA).

### RNA Isolation and Quantitative RT-PCR

RNA was prepared from primary tissues or extracted from paraffin-embedded sections using the Tizol or QIAamp DNA FFPE Tissue Kit (Qiagen, Valencia, CA, USA). cDNA was synthesized using Superscript II (Invitrogen) and oligo (dT)12-18 primers. Real-time RT-PCR was performed using SYBR green (Takara, Shiga, Japan) in ABI PRISM 7000 (Applied Biosystems, Foster City, CA, USA). All expression values were normalized to β*-Actin* or *Gapdh* mRNA levels.

### Primer Sequences for Quantitative RT-PCR

mouse *Zo-1*-F, 5′-CCCCTCTGTCCAGCTCTTC-3′; mouse *Zo-1*-R,5′-CACCGGAGTGATGGTTTTCT-3′; mouse *Occludin*-F, 5′-CCTCCAATGGCAAAGTGAAT-3′; mouse *Occludin*-R, 5′-CTCCCCACCTGTCGTGTAGT-3′; mouse *Claudin 1*-F, 5′-TGG GTT TCA TCC TGG CTT CT-3′; mouse *Claudin 1*-R, 5′-TGT ATC TGC CCG GTG CTT T-3′; mouse *Gapdh*-F, 5′-AGGTCGGTGTGAACGGATTTG-3′; mouse *Gapdh*-R, 5′-TGTAGACCATGTAGTTGAGGTC-3′; mouse β*-Actin*-F, 5′-AGGTGACAGCATTGCTTCTG-3′; mouse β*-Actin*-R, 5′-GCTGCCTCAACACCTCAAC-3′.

### Statistical Analysis

All values are expressed as the means ± SE. The incidence of diabetes was plotted as the Kaplan-Meier curve and compared using the logrank test. Two-tailed Student's *t*-test was employed to compare values between two groups. *P*-values < 0.05 were considered significant.

## Results

### Reduced Incidence of Diabetes by Probiotic Combination

**Figure 1:**
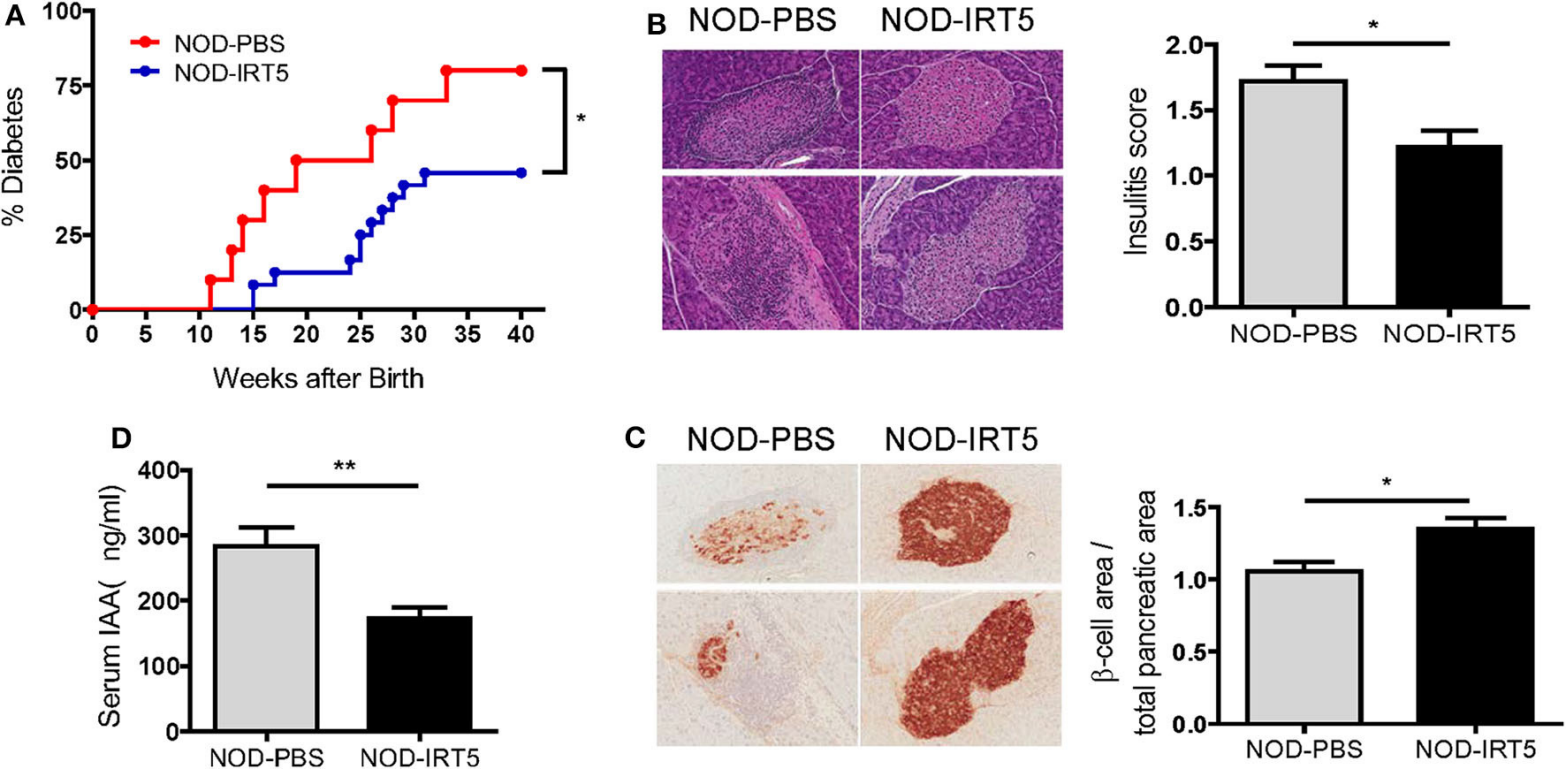
Incidence of diabetes in female NOD mice. **(A)** IRT5 was administered since 4 weeks of age for 36 weeks, and the incidence of diabetes was monitored. Control group was treated with solvent only in the same manner (*n* = 10 for NOD-PBS and 24 for NOD-IRT5). **(B,C)** Changes of pancreatic islets. Insulitis score **(B)** and relative β-cell mass **(C)** were determined after 12 weeks of IRT5 administration as described in the MATERIALS AND METHODS (*n* = 8 for NOD-PBS and 9 for NOD-IRT5 in B; *n* = 7 for NOD-PBS and 8 for NOD-IRT5 in **C**). **(D)** Serum level of insulin autoantibody (IAA) was determined in NOD-PBS and NOD-IRT5 using ELISA (*n* = 6 for NOD-PBS and 6 for NOD-IRT (**p < 0.01; *p < 0.05).

Since IRT5, a combination of 5 bacteria (*Lactobacillus acidophilus, Lactobacillus casei, Lactobacillus reuteri, Bifidobacterium bifidium*, and *Streptococcus thermophilus*) has been shown to be effective against several autoimmune and inflammatory disorders (20, 21), we administered 5 × 10^8^ cfu IRT5 to female NOD mice by oral gavage six times a week for 36 weeks beginning at 4 weeks of age and monitored blood glucose level. The incidence of diabetes was significantly reduced in NOD mice treated with IRT5 compared to control NOD mice treated with PBS (Figure 1A). Consistent with the reduced incidence of diabetes, insulitis score was also significantly reduced in mice treated with IRT5 (Figure 1B). Conversely, β-cell mass was higher in mice treated with IRT5 compared to control NOD mice (Figure 1C). Serum level of IAA, an index of autoimmunity in NOD mice (26), was also reduced in NOD mice treated with IRT5 compared to control mice (Figure 1D).

### Reduced Gut Permeability by Probiotic Combination

**Figure 2:**
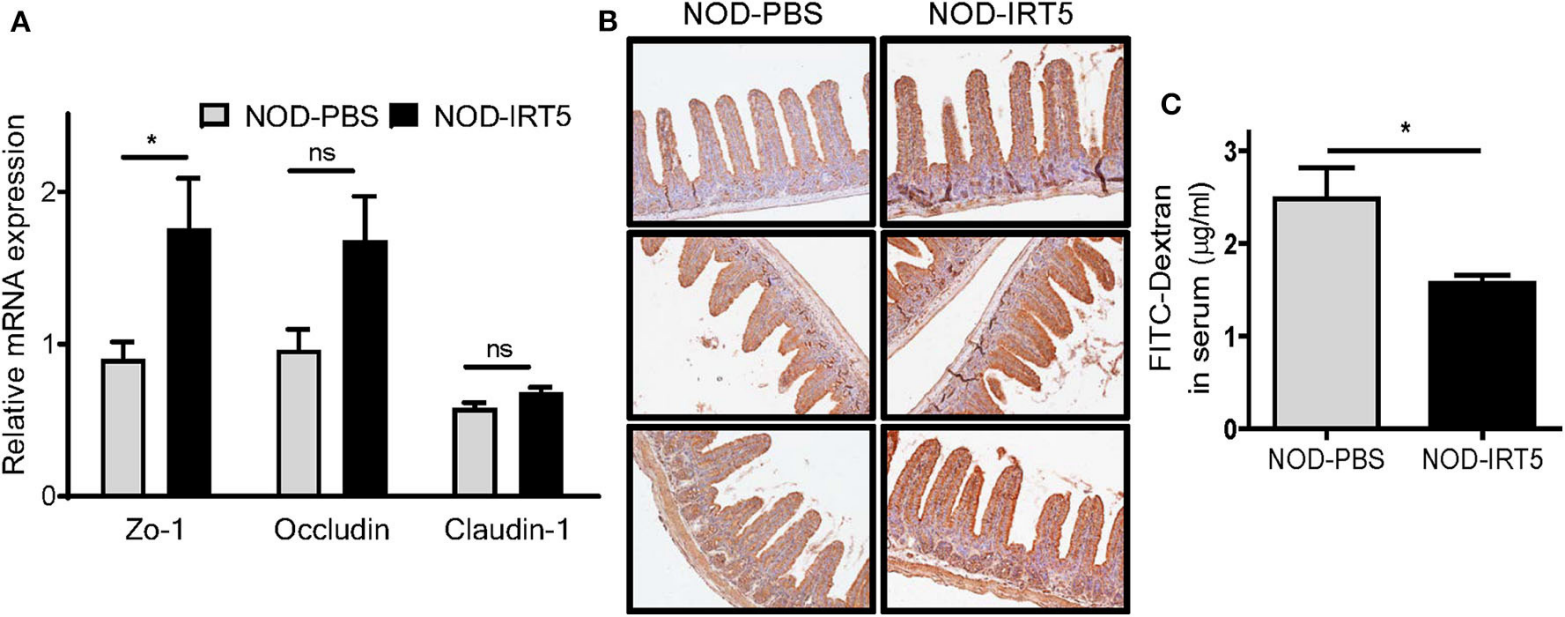
Changes of gut permeability in NOD mice treated with IRT5. **(A)** The expression of tight junction proteins was evaluated by real-time RT-PCR using mRNA from paraffin-embedded tissues and specific primers (*n* = 7 for NOD-PBS and 5 for NOD-IRT5). **(B)** The expression of ZO-1 protein was evaluated by immunohistochemistry. **(C)** Gut permeability was determined by measuring serum FITC fluorescence 4 h after oral administration of FITC-dextran (*n* = 5 for NOD-PBS and 5 for NOD-IRT5) (*p < 0.05; ns, not significant).

To study the mechanism of the reduced incidence of autoimmune diabetes by IRT5, we studied possible changes of permeability of gut epithelium since the intestine is the first organ contacting oral administered probiotics and gut permeability is changed in patients with T1D or animal models of autoimmune diabetes, potentially contributing to the acceleration of islet autoimmunity (27–29). Real-time RT-PCR showed that the expression of *Zo-1* encoding a tight junction protein that constitutes critical physical barrier of the intestine, was significantly increased in the SI of NOD mice treated with IRT5 for 12 weeks (Figure 2A), suggesting improved gut barrier function. The expression of *Occludin* encoding another tight junction protein, was also increased after IRT5 administration for 12 weeks, while statistical significance was not achieved (Figure 2A). In contrast, the expression of *Claudin-1* that controls epithelial permeability as a tight junction protein (30) was not significantly changed after IRT5 treatment (Figure 2A). Significantly increased expression of ZO-1 after IRT5 treatment was confirmed by immunohistochemistry (Figure 2B). When we investigated the intestinal barrier function of gut epithelium by measuring the amount of circulating FITC-dextran 4 h after oral gavage with FITC-dextran, a significantly reduced serum content of FITC-dextran was observed in mice treated with IRT5 for 12 weeks compared to control NOD mice (Figure 2C), supporting enhanced barrier function by IRT5.

### Increased Gut-Homing Treg Cells by Probiotic Combination

We next studied changes of immune parameters involved in β-cell autoimmunity after administration of IRT5. We first assessed priming of diabetogenic T cells using the BDC2.5 TCR transgenic system that occurs in PLNs and is critical in the sensitization of diabetogenic T cells *in vivo* (29). The transgene-encoded TCR, derived from a diabetogenic BDC2.5 T cell clone, recognizes chromogranin A as a β-cell autoantigen in the context of MHC class II molecule I-A^g7^ (31). We observed no significant change of the proliferation of transferred BDC2.5 T cell in NOD mice treated with IRT5 (Figure S2), indicating that IRT5 administration does not influence diabetogenic T cell priming in PLNs.

**Figure 3:**
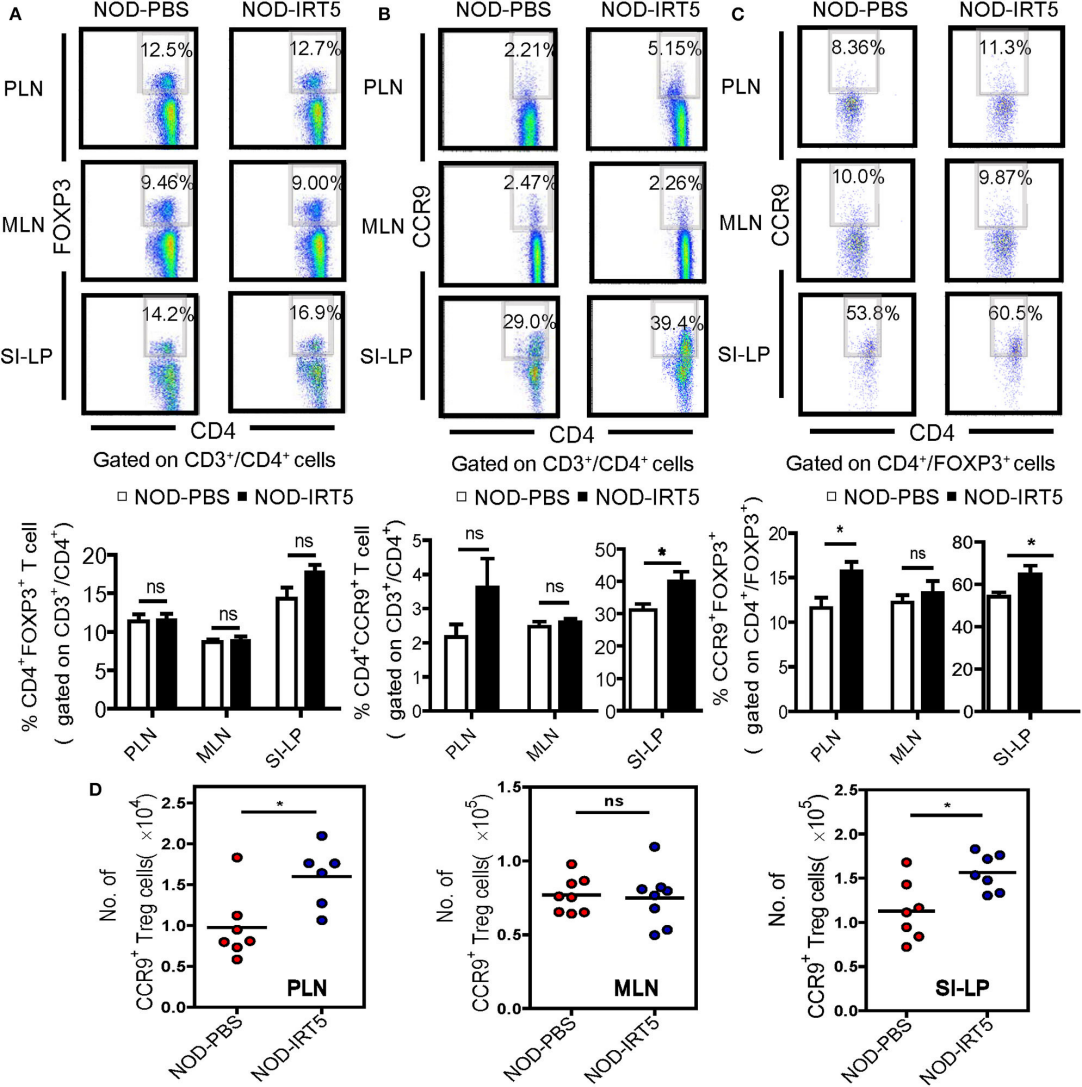
Treg cells in NOD mice treated with IRT5. **(A)** Cells from pancreatic lymph nodes (PLNs), mesenteric lymph nodes (MLNs), or lamina propria of the small intestine (SI-LP) of NOD mice that were treated with IRT5 for 12 weeks were stained with anti-CD4 and -FOXP3 mAbs for flow cytometry gated on CD3 and CD4. The percentages of CD4^+^FOXP3^+^ cells among CD3^+^CD4^+^ cells were compared (bottom). Representative scattergrams are shown (top). The numbers in the top right quadrants represent the percentage of CD4^+^FOXP3^+^ Treg cells among CD3^+^CD4^+^ cells analyzed (*n* = 6 for NOD-PBS and 7 for NOD-IRT5 in PLNs; *n* = 6 for NOD-PBS and 7 for NOD-IRT5 in MLNs; *n* = 9 for NOD-PBS and 9 for NOD-IRT5 in SI-LP). **(B)** Cells prepared as in **(A)** were stained with anti-CD4 and -CCR9 mAbs for flow cytometry gated on CD3 and CD4. The percentages of CD4^+^CCR9^+^ cells among CD3^+^CD4^+^ cells were compared (bottom). Representative scattergrams are shown (top). The numbers in the top right quadrants represent the percentage of CD4^+^CCR9^+^ Treg cells among CD3^+^CD4^+^ cells analyzed (*n* = 8 for NOD-PBS and 8 for NOD-IRT5 in PLNs; *n* = 10 for NOD-PBS and 8 for NOD-IRT5 in MLNs; *n* = 7 for NOD-PBS and 7 for NOD-IRT5 in SI-LP). **(C)** Cells prepared as in **(A)** were stained with anti-FOXP3 and -CCR9 mAbs for flow cytometry gated on CD4 and FOXP3. The percentages of CCR9^+^FOXP3^+^ cells among CD4^+^FOXP3^+^ cells were compared (bottom). Representative scattergrams are shown (top). The numbers in the top right quadrants represent the percentage of CCR9^+^FOXP3^+^ Treg cells among CD4^+^FOXP3^+^ cells analyzed (*n* = 7 for NOD-PBS and 12 for NOD-IRT5 in PLNs; *n* = 7 for NOD-PBS and 12 for NOD-IRT5 in MLNs; *n* = 9 for NOD-PBS and 10 for NOD-IRT5 in SI-LP). **(D)** The numbers of CCR9^+^CD4^+^FOXP3^+^ cells were calculated from the total numbers of lymphocytes, the percentages of CD3^+^CD4^+^ cells among total lymphocytes and that of CCR9^+^FOXP3^+^ cells among CD3^+^CD4^+^ cells (*p < 0.05; ns, not significant).

To assess other immune parameters modulating β-cell autoimmunity, we studied Treg cells that are induced by IRT5 and can exert inhibitory effects on the development of autoimmune diabetes (32, 33), together with T cell subsets. The proportions of CD3^+^, CD4^+^, or CD8^+^ T cells were not significantly changed in PLNs, MLNs or SI-LP after IRT5 administration (Figure S3). Contrary to our expectation, the proportion of CD4^+^FOXP3^+^ Treg cells was not significantly changed in PLNs or MLNs after IRT5 administration for 12 weeks (Figure 3A). There was an increasing tendency of CD4^+^FOXP3^+^ Treg cells in SI-LP of the treated mice; however, the difference was statistically insignificant (Figure 3A). We next studied the expression of CCR9 that can be expressed in Treg cells and is required for T cell homing to the SI (34, 35). We observed increased expression of CCR9 in CD4^+^ T cells from SI-LP (Figure 3B). The expression of CCR9 in CD4^+^ T cells was not significantly changed in PLNs or MLNs after IRT5 administration, while there was an increasing tendency of CCR9 in CD4^+^ T cells of PLNs (Figure 3B). When we studied the changes of CCR9^+^ Treg cells, the proportion of CCR9^+^CD4^+^FOXP3^+^ Treg cells was significantly increased in PLNs and SI-LP but not in MLNs after IRT5 administration (Figure 3C), suggesting the role of gut-homing Treg cells in the reduced incidence of autoimmune diabetes by IRT5. To confirm the increases of CCR9^+^CD4^+^FOXP3^+^ Treg cells after IRT5 treatment, we calculate the numbers of CCR9^+^CD4^+^FOXP3^+^ Treg cells in PLNs, MLNs or SI-LP. Indeed, the numbers of CCR9^+^CD4^+^FOXP3^+^ Treg cells were significantly increased in PLNs and SI-LP but not in MLNs of NOD mice treated with IRT5 (Figure 3D), corroborating the increases of CCR9^+^CD4^+^FOXP3^+^ Treg cells after IRT5 administration in PLNs and SI-LP.

### Reduced Th1 Polarization by IRT5

**Figure 4:**
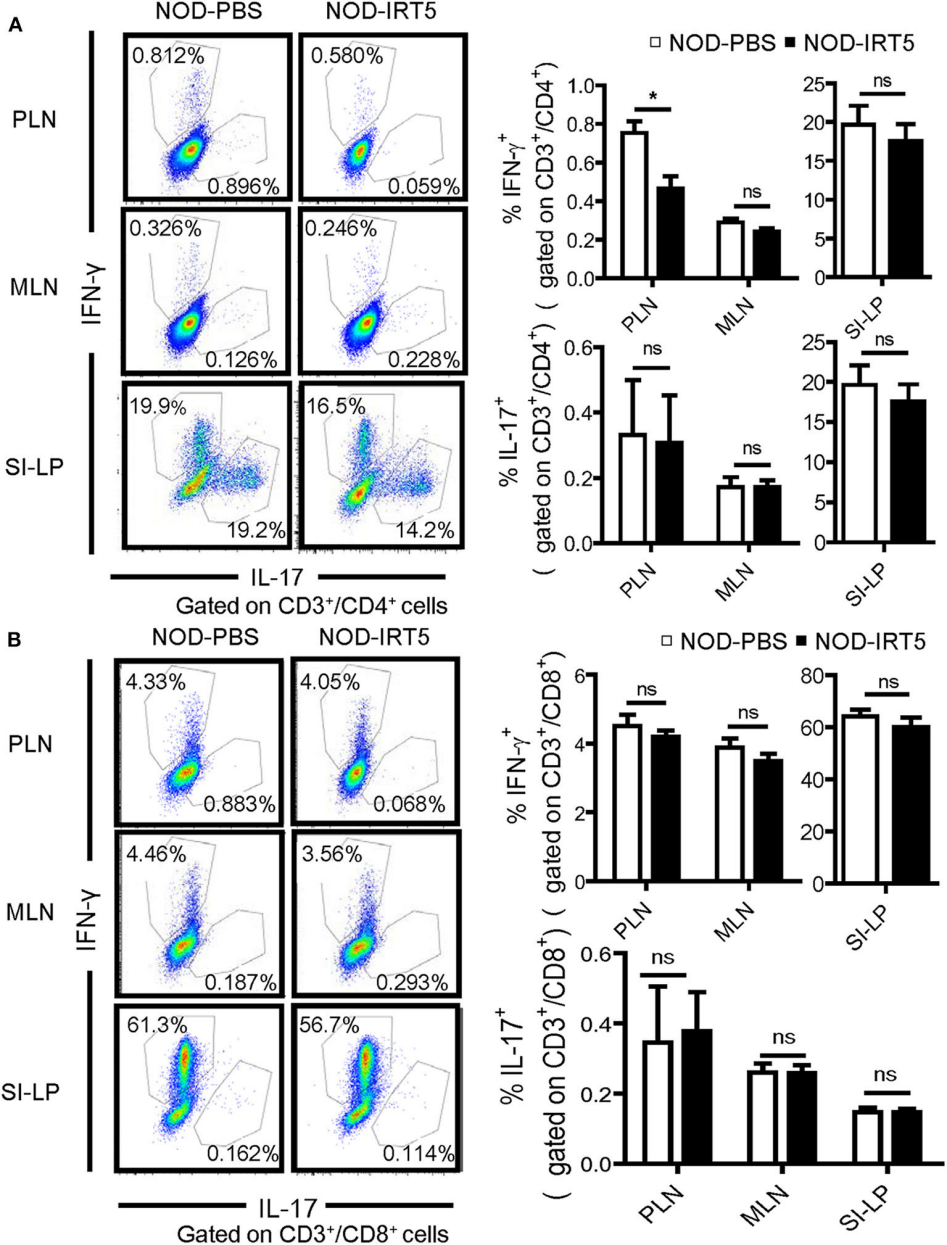
Th1/Th17 skewing in NOD mice treated with IRT5 for 12 weeks. **(A)** CD4^+^ Th cell skewing in PLNs, MSLs, or SI-LP was evaluated by flow cytometry. The proportions of IFN-γ^+^ cells and IL-17^+^ cells among CD3^+^CD4^+^ T cells (right). Representative scattergrams are shown (left) (*n* = 5 for NOD-PBS and 5 for NOD-IRT5 in PLNs; *n* = 5 for NOD-PBS and 4 for NOD-IRT5 in MLNs; *n* = 5 for NOD-PBS and 5 for NOD-IRT5 in SI-LP). **(B)** CD8^+^ Tc cell skewing in PLNs, MSLs, or SI-LP was evaluated by flow cytometry. The proportions of IFN-γ^+^ cells and IL-17^+^ cells among CD3^+^CD8^+^ T cells (right). Representative scattergrams are shown (left) (*n* = 5 for NOD-PBS and 5 for NOD-IRT5 in PLNs; *n* = 5 for NOD-PBS and 5 for NOD-IRT5 in MLNs; *n* = 5 for NOD-PBS and 5 for NOD-IRT5 in SI-LP) (*p < 0.05; ns, not significant).

We next studied whether IRT5 administration can change the polarization of Th cells which affects the development of autoimmune diabetes at the effector phase (36). We observed that IRT5 administration significantly reduced the proportion of CD4^+^IFN-γ^+^ Th1 cells in PLNs but not in MLNs or SI-LP (Figure 4A). The proportion of CD4^+^IL-17A^+^ Th17 cells was not significantly changed after IRT5 administration in PLNs, MLNs or SI-LP (Figure 4A). We also studied skewing of CD8^+^ T cells since CD8^+^ T cells are critical effector cells in autoimmune diabetes and CD8^+^ T cells can be divided into multiple subsets according to cytokine profile, similar to CD4^+^ T cells (37). The proportions of CD8^+^IFN-γ^+^ Tc1 cells or CD8^+^IL17-A^+^ Tc17 cells were not significantly changed after IRT5 administration in PLNs, MLNs or SI-LP (Figure 4B), suggesting that IRT5 inhibits the development of autoimmune diabetes by modulating skewing of CD4^+^ T cells rather than that of CD8^+^ T cells.

## Discussion

We observed a significant decrease of diabetes incidence after IRT5 administration for 36 weeks, which was associated with reduced gut permeability and increased proportion of CCR9^+^ gut-tropic Treg cells. In this investigation, we started IRT5 administration at 4 weeks of age because previous studies showed that inhibition of physiological β-cell apoptosis at day 14–42 with benzyloxylcarbonyl-V-A-d-*O*-methyl fluoromethyl ketone (zVAD-fmk), a pan-caspase inhibitor, was able to inhibit sensitization to diabetogenic T cells (38) and immune intervention at 4 weeks of age could block the development of diabetes in NOD mice (39–41). We continued IRT5 treatment up to 40 weeks of age because diabetes developed even after 32–36 weeks of age in our NOD colonies (25). The reduction of gut permeability by IRT5 is consistent with previous papers showing decreased gut permeability by several *Lactobacilli* in diverse conditions (42). The pathogenic role of increased gut permeability due to dietary cause is widely accepted in type 2 diabetes (43, 44). On the other hand, the mechanism and immunological impact of altered gut permeability in autoimmune diabetes are less clear. However, several papers have shown increased gut permeability in T1D patients or animal models of autoimmune diabetes (27–29). Morphological changes of gut epithelium, such as reduced crypt length were observed after administration of *Akkermansia* to NOD, which was accompanied by delayed onset of diabetes (45). Alteration in dietary components, gut bacteria, inflammation or other factors may contribute to the increased gut permeability in autoimmune diabetes, which is likely to affect immune tolerization or other immune processes in gut epithelium, gut-associated lymphoid tissues or PLNs, influencing autoimmune processes (46).

When we studied the immunological impact of IRT5 administration, we observed no significant change in the proportion of total Treg cells in PLNs, MLNs or SI-LP after *in vivo* administration for 12 weeks. Such results are in contrast to a significant induction of Treg cells in MLNs after IRT5 administration for 20 days (21), which could be due to differences in disease models. Prolonged administration of IRT5 might lead to the downregulation of immunological changes associated with probiotics administration. Difference in other experimental conditions, such as distinct mouse strain might be the cause of discrepant results between studies.

Instead, we observed a significant increase in the percentage of CCR9^+^ Treg cells, indicating that the proportion of Treg cells with gut-homing capability was increased by IRT5 administration *in vivo*. Since CCR9, a gut-homing receptor, is expressed in Treg cells and crucial in Treg cell homing to gut tissue (35), an increased proportion of CCR9^+^ Treg cells after IRT5 administration is likely to enhance Treg cell homing to target tissues, such as the intestine or pancreas. In contrast, a previous paper reported that a butyrate-producing probiotics (*Clostridium butyricum* CB0313.1) increases total Treg cells in PLNs with significant protection against autoimmune diabetes of NOD mice (7). Nonetheless, the induction of Treg cells with α4β7 gut-homing receptor was particularly prominent, suggesting the importance of gut-homing Treg cells in probiotics-induced protection against autoimmune diabetes (7). Despite significant induction of Treg cells expressing CCR9, we observed no effect of IRT5 administration on diabetogenic T cell priming in PLNs, draining LNs for T cells reactive to islet β-cell antigens. Such a failure to suppress diabetogenic T cell priming in PLNs by Treg with gut-homing receptor is probably because Treg cells act in target organs but not in draining LNs (32). CCR9^+^ T cells interact with its ligand, CCL25, to enter target organs, and CCR9^+^ Treg cells would exert their effect after homing to target tissues through interaction with its ligand, which can explain the absence of reduced diabetogenic T cell priming in PLNs by IRT5. Consistent with the notion that Treg cells act in target tissue, the decrease of the Treg cell:T effector cell ratio was observed in pancreatic islets but not in PLNs of NOD mice (47). Increased FOXP3^+^ cell number has also been found in pancreatic islets after the treatment of NOD mice with *Akkermansia*, which was accompanied by delayed development of diabetes (45). Significant increases of CCR9^+^ Treg cells in SI-LP observed in this study also support the role of CCR9 on gut homing and potential effect of CCR9^+^ Treg cells in target tissues. No significant changes of total Treg cells in PLNs might also contribute to the absence of IRT5 effect on diabetogenic T cell priming in PLNs.

Th1 polarization is an important step in the pathogenesis of autoimmune diabetes in NOD mice (36). Furthermore, IRT5 can downregulate Th1/Th17 polarization through generation of regulatory DCs and subsequent induction of Treg cells (20). We observed reduced skewing to Th1 producing IFN-γ in PLNs by IRT5 administration, and similar changes were observed in MLNs or SI-LP but without statistical significance. Since Th1 cells producing IFN-γ are main effector cells in autoimmune diabetes (36, 48), reduced Th1 skewing by IRT5 is likely to contribute to the reduced incidence of autoimmune diabetes by IRT5. Significant changes of the proportion of Th1 cells in PLNs without alteration of diabetogenic T cell priming by IRT5 treatment could be due to differences in signal transduction and proliferative response between naïve cells and primed T cells (49, 50). Recirculation of effector T cells back and forth between target tissues and draining lymph nodes (51, 52) might also contribute to the reduced Th1 cells in PLNs because CCR9^+^ Treg cells are likely to act on effector T cells in pancreatic islets after homing to target tissues and such effector T cells may move back to PLNs. In contrast to the skewing to Th1, skewing to Th17 producing IL-17A was not changed by IRT5 administration, while the role of Th17 cells in autoimmune diabetes begins to be recognized (53). Although the effect of IRT5 reducing the incidence of autoimmune diabetes of NOD mice is most likely due to induction of gut-homing Treg cells and reduced Th1 polarization, other mechanisms have been suggested, such as restoration of antigen-specific tolerance or induction of IL-10 when genetically-modified *Lactococcus lactis* or a probiotic combination consisting of *Bifidobacteria, Lactobacilli*, and *Streptococcus salivarius subsp. Thermophiles* was employed (6, 54).

In conclusion, we observed significantly reduced incidence of autoimmune diabetes in NOD mice by administration of a probiotic combination comprising 5 bacteria, which can be explained by reduced gut permeability, increased generation of gut-homing Treg cells and reduced Th1 polarization. However, autoimmune diabetes of NOD mice was not completely abrogated by our probiotic combination, which could be a drawback in the clinical application of IRT5 to the prevention or treatment of autoimmune diabetes. Further optimization of IRT5 by combining with other bacteria or their products comprising active components may lead to the discovery of effective therapeutic or preventive compounds that can be safely used for human trial or clinical purposes. Because we recently identified cell surface β-glucan/galactan polysaccharide of *Bifidobacterium bifidum* as an active molecule inducing Treg cells (55), IRT5 or their active components could be candidates of therapeutic agents for human immune disorders in future studies.

## Data Availability Statement

All datasets generated for this study are included in the article/Supplementary Material.

## Ethics Statement

The animal study was reviewed and approved by IACUC of the Department of Laboratory Animal Resources of Yonsei University College of Medicine, an AAALAC-accredited unit.

## Author Contributions

M-SL and S-HI conceived and designed the experiment. TK and J-CL conducted the experiment. All authors contributed to the drafting of the manuscript.

## Conflict of Interest

J-CL and S-HI are employed by company ImmunoBiome. Inc. The remaining authors declare that the research was conducted in the absence of any commercial or financial relationships that could be construed as a potential conflict of interest.
