# Supplementary material for: Amelioration of Autoimmune Diabetes of NOD Mice by Immunomodulating Probiotics
Source: Front Immunol. 2020 Sep 3;11:1832. doi: 10.3389/fimmu.2020.01832 (PMC7496355; doi:10.3389/fimmu.2020.01832)
Supplement: Supplementary file 1 [file Presentation_1.PPTX]

## Slide 1
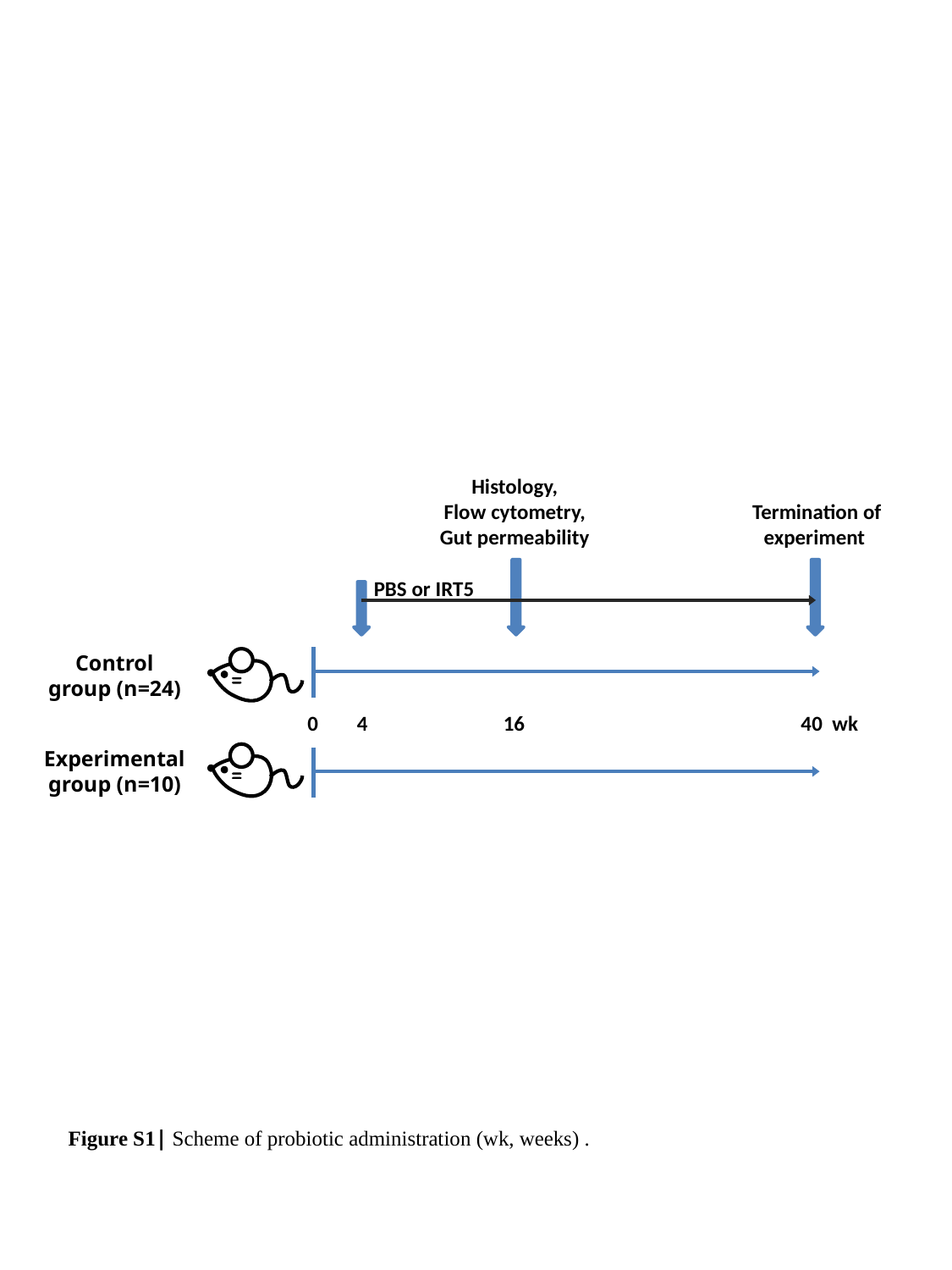

Histology,
Flow cytometry,
Gut permeability
Termination of experiment
PBS or IRT5
Control
group (n=24)
 0 4 16 40 wk
Experimental
group (n=10)
Figure S1| Scheme of probiotic administration (wk, weeks) .

## Slide 2
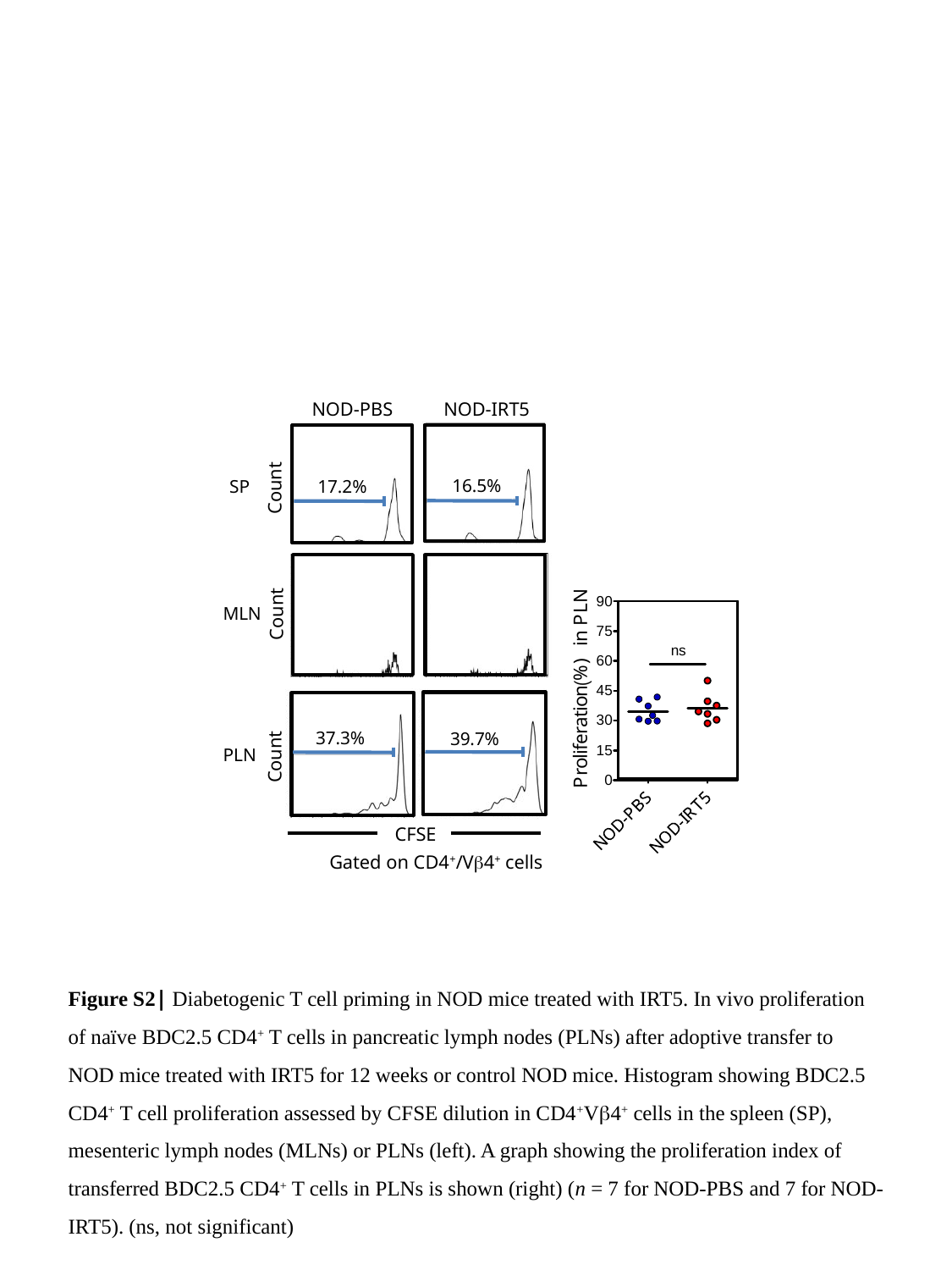

NOD-PBS
NOD-IRT5
SP
16.5%
17.2%
Count
MLN
Count
PLN
37.3%
39.7%
Count
CFSE
Gated on CD4+/Vb4+ cells
Figure S2| Diabetogenic T cell priming in NOD mice treated with IRT5. In vivo proliferation of naïve BDC2.5 CD4+ T cells in pancreatic lymph nodes (PLNs) after adoptive transfer to NOD mice treated with IRT5 for 12 weeks or control NOD mice. Histogram showing BDC2.5 CD4+ T cell proliferation assessed by CFSE dilution in CD4+Vβ4+ cells in the spleen (SP), mesenteric lymph nodes (MLNs) or PLNs (left). A graph showing the proliferation index of transferred BDC2.5 CD4+ T cells in PLNs is shown (right) (n = 7 for NOD-PBS and 7 for NOD-IRT5). (ns, not significant)

## Slide 3
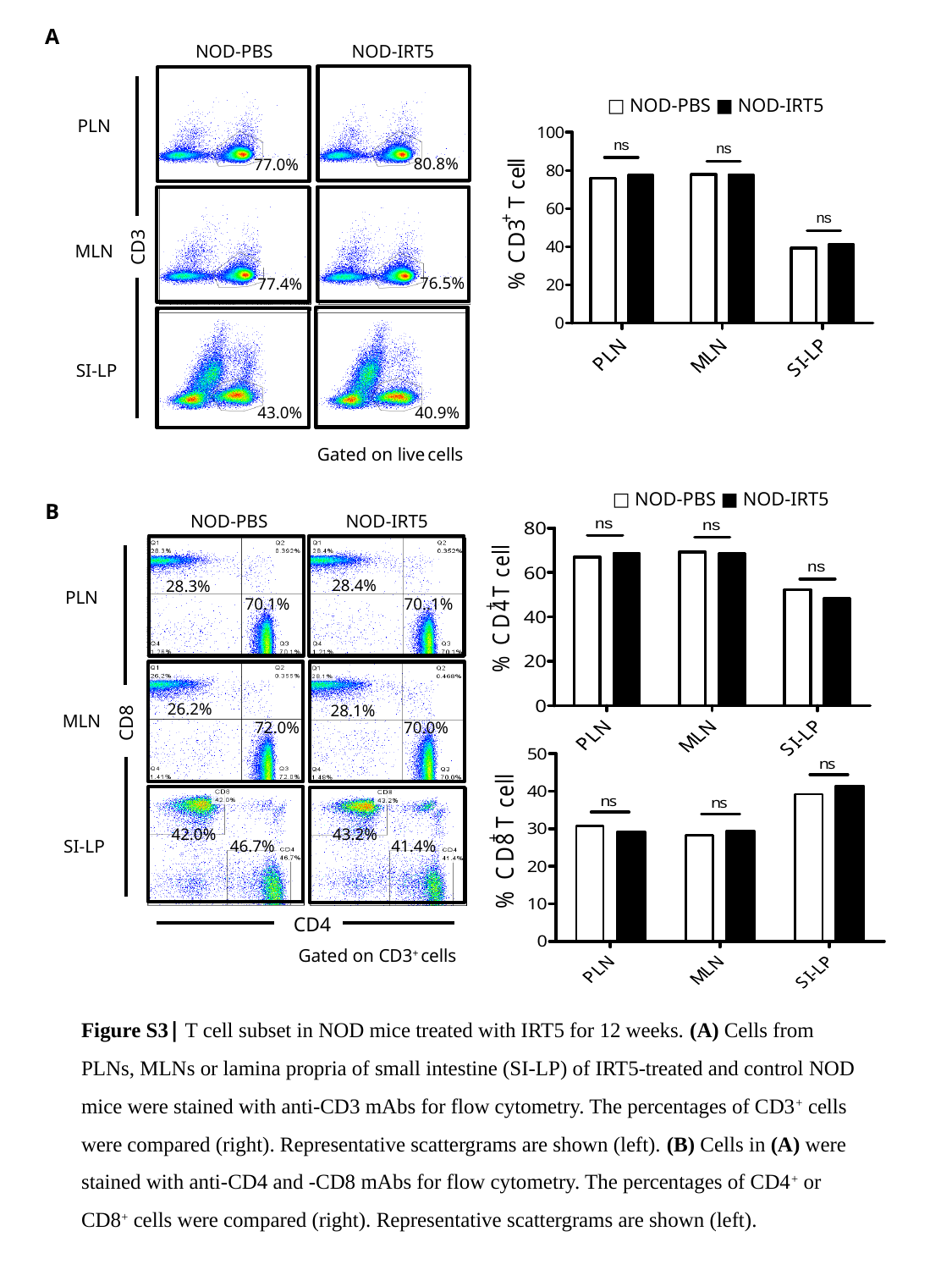

A
NOD-PBS
NOD-IRT5
80.8%
77.0%
□ NOD-PBS ■ NOD-IRT5
PLN
76.5%
77.4%
CD3
MLN
40.9%
43.0%
SI-LP
Gated on live cells
□ NOD-PBS ■ NOD-IRT5
B
NOD-PBS
NOD-IRT5
28.4%
28.3%
70.1%
70..1%
PLN
26.2%
28.1%
72.0%
70.0%
MLN
CD8
42.0%
43.2%
46.7%
41.4%
SI-LP
CD4
Gated on CD3+ cells
Figure S3| T cell subset in NOD mice treated with IRT5 for 12 weeks. (A) Cells from PLNs, MLNs or lamina propria of small intestine (SI-LP) of IRT5-treated and control NOD mice were stained with anti-CD3 mAbs for flow cytometry. The percentages of CD3+ cells were compared (right). Representative scattergrams are shown (left). (B) Cells in (A) were stained with anti-CD4 and -CD8 mAbs for flow cytometry. The percentages of CD4+ or CD8+ cells were compared (right). Representative scattergrams are shown (left).
